# A Rare Etiology of Peripheral Facial Paralysis in the Emergency Department: A Case Report

**DOI:** 10.7759/cureus.90089

**Published:** 2025-08-14

**Authors:** Esmeralda Hoxhaj, Antoine Baudrez, Céline Couvreur

**Affiliations:** 1 Internal Medicine, Centre Hospitalier Universitaire (CHU) Université Catholique de Louvain (UCLouvain) Namur, Godinne, BEL; 2 Radiology, Centre Hospitalier Universitaire (CHU) Université Catholique de Louvain (UCLouvain) Namur, Godinne, BEL; 3 Emergency Medicine, Centre Hospitalier Universitaire (CHU) Université Catholique de Louvain (UCLouvain) Namur, Godinne, BEL

**Keywords:** case report, facial nerve palsy, hemiparesis, millard-gubler syndrome, pontine infarct

## Abstract

Peripheral facial paralysis is a common reason for emergency department visits. It is distinguished from central facial paralysis by the involvement of the entire musculature of the affected hemiface. In most cases, it is benign and caused by an infection. In the emergency department, it is typically triaged as a minor emergency. However, in rare cases, it may be part of Millard-Gubler syndrome (MGS), resulting from a vascular lesion, most notably a stroke, affecting the pons. The following case describes a patient who presented with peripheral facial paralysis, later diagnosed as a pontine ischemic stroke. It aims to raise awareness among emergency physicians about the risk of prematurely dismissing peripheral facial paralysis as benign.

## Introduction

Peripheral facial paralysis is defined as a lesion of the facial nerve after its exit from the brainstem. It presents clinically as muscle weakness of the hemiface on the same side as the lesion. There are no other associated neurological deficits, and the etiology is most often benign, with a generally favorable outcome [1]. 

Millard-Gubler syndrome (MGS) is a crossed brainstem syndrome resulting from a lesion of the ventral part of the pons. It is characterized by ipsilateral peripheral facial paralysis (due to involvement of the facial nerve nucleus) and contralateral hemiparesis (due to involvement of the pyramidal tract crossing the pons). In most cases, it is secondary to an ischemic or hemorrhagic stroke [2]. 

We present here the case of a patient admitted to the emergency department for peripheral facial paralysis, in whom a central cause was revealed by imaging. This case emphasizes the critical need to recognize peripheral facial paralysis as a potentially serious symptom and to systematically assess for additional neurological deficits in the triage process. 

## Case presentation

**Figure 1:**
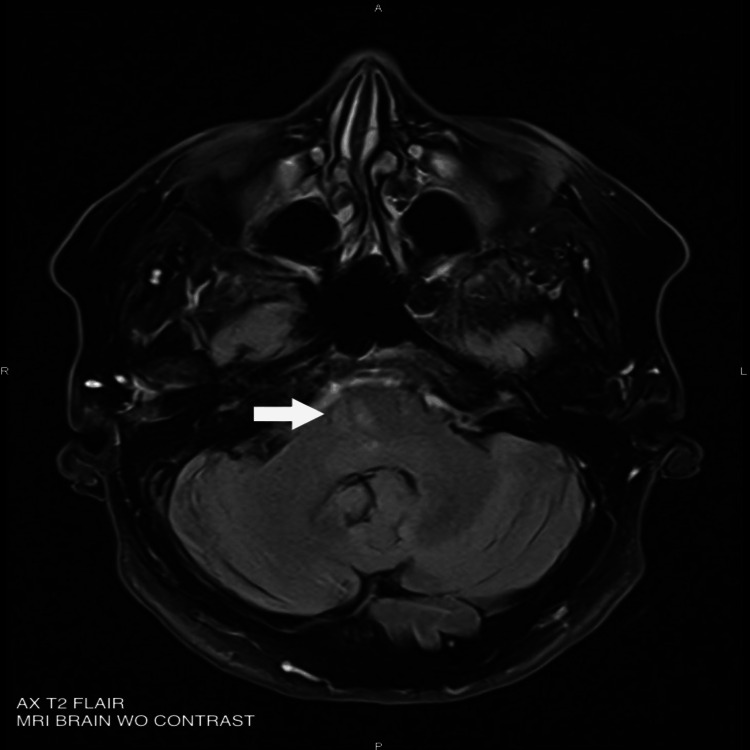
Brain MRI of the patient White arrow: Hyperintense lesion on FLAIR sequence in the right inferolateral pons; FLAIR: Fluid-attenuated inversion recovery; AX: Axial; WO: Without

**Figure 2:**
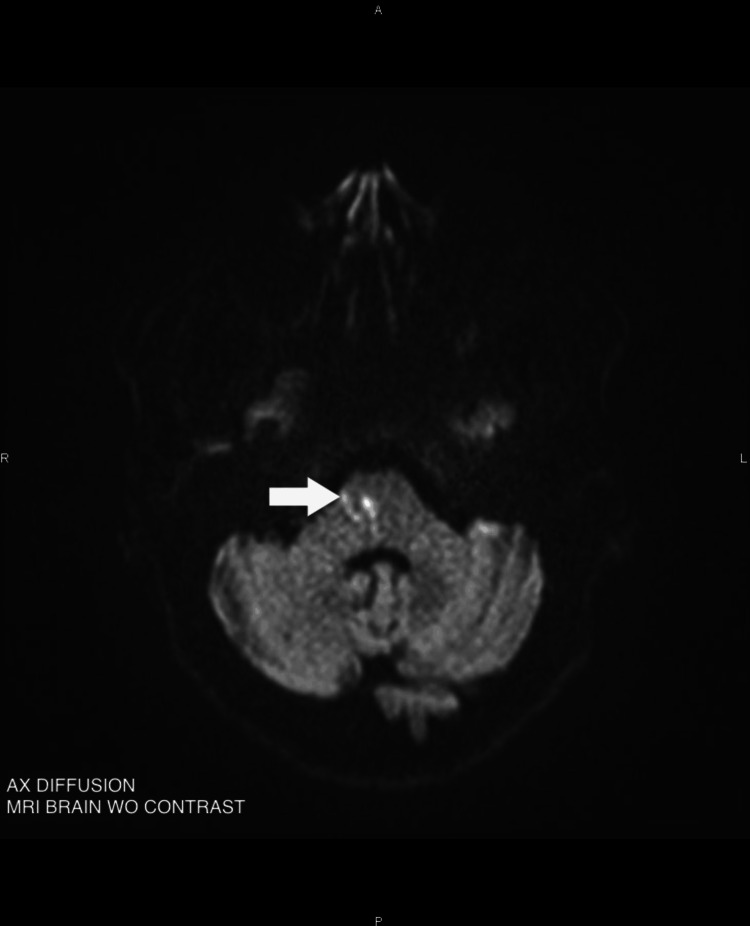
Brain MRI White arrow: Hyperintense lesion on diffusion sequence in the right inferolateral pons; AX: Axial; WO: Without

We report the case of a 59-year-old woman admitted to the emergency department for facial paralysis since awakening. Her medical history includes pulmonary embolism, dyslipidemia, obesity, depression, and former tobacco use. Her chronic medications include rosuvastatin/ezetimibe and escitalopram. Upon arrival at the emergency department, her blood pressure was 162/80 mmHg, heart rate was 83 beats per minute, oxygen saturation was 97% on room air, and blood glucose was 6.22 mmol/L. The patient was afebrile. On admission, the triage nurse noted a right peripheral facial paralysis with both the upper and lower parts of the face involved. She was triaged as a minor emergency, and blood tests, including viral serologies, were performed. However, during a more thorough neurological examination, the patient was found to have a subtle left-sided hemiparesis, with muscle strength graded as four out of five on the Medical Research Council (MRC) scale. Given this atypical clinical presentation, a brain CT scan was performed, which showed no lesion explaining the symptoms. Brain MRI, however, revealed an ischemic stroke in the inferolateral part of the pons, as seen in Figures 1-2.

Due to a low National Institutes of Health Stroke Scale (NIHSS) score of three, the patient was not eligible for thrombolysis. Treatment with 250 mg of intravenous aspirin and 300 mg of oral clopidogrel was initiated, and the patient was admitted to the stroke unit for management. She was able to return home a few days later while continuing rehabilitation physiotherapy sessions for persistent weakness of the left side of the body.

## Discussion

When managing a facial paralysis in the emergency department, the first step is to distinguish between a central facial paralysis and a peripheral facial paralysis. Peripheral facial paralysis is due to a lower motor neuron lesion and presents as a motor deficit of all ipsilateral facial muscles on the side of the facial nerve lesion. Central facial paralysis is due to an upper motor neuron lesion and causes a motor deficit of the lower facial muscles (visible at the mouth) contralateral to the lesion in the cortex or, if the brainstem is affected, above the pons [3]. This distinction can be easily made by assessing the patient's ability to smile and to close both eyes [1]. Our patient showed involvement of both the upper and lower facial muscles, confirming peripheral facial paralysis.

The severity of peripheral facial paralysis is graded using the House-Brackmann scale [4]. The etiologies of peripheral facial paralysis are diverse, with infection being the leading cause (viral infections, including herpes, acute otitis media, and Lyme disease). Less common causes include sarcoidosis or neoplasia [1]. Treatment of peripheral facial paralysis is based on oral corticosteroids, initiated preferably within the first three days after symptom onset. Corticosteroids improve the rate and the rapidity of motor recovery if started within 72 hours of symptom onset [5,6]. In severe cases of peripheral facial paralysis, the addition of valacyclovir to corticosteroids has demonstrated a higher rate of complete recovery [7]. However, only about 70% of patients fully regain mobility after treatment [8].

Millard-Gubler syndrome was first described in 1858 by Auguste Louis Jules Millard and Adolphe-Marie Gubler. It results from a unilateral lesion of the basal portion of the ventral pons. Clinically, it presents as an ipsilateral facial nerve (cranial nerve VII) palsy combined with involvement of the corticospinal tract in the pons above the medullary pyramidal decussation. Diagnosis is thus based on the association of peripheral facial paralysis and contralateral hemiparesis affecting both the upper and lower limbs. Foville syndrome is almost similar to MGS but also includes ipsilateral abducens nerve (cranial nerve VI) palsy as part of the core clinical picture. Given the proximity of numerous tracts to the facial nerve nucleus, MGS is often associated with additional neurological deficits such as contralateral cerebellar ataxia or contralateral hemibody paresthesias [2].

The etiology of this syndrome varies with age [2]. In younger patients, causes may include infectious conditions such as viral rhombencephalitis, tuberculosis, or neurocysticercosis [9-11]; neoplasms; demyelinating disorders like multiple sclerosis; and autoimmune conditions such as Behçet’s disease. In these cases, the onset of symptoms is generally more gradual [2,12].

In older patients, cerebrovascular accidents involving the posterior circulation are the most frequent cause. These events may be hemorrhagic, such as a prepontine subarachnoid hematoma or hemorrhage secondary to a cavernous vascular malformation of the brainstem compressing pontine arterial structures [13], or ischemic, resulting from occlusion of a short circumferential branch of the basilar artery [14].

Ischemic strokes account for 85% of all strokes. Those affecting the brain stem are uncommon, accounting for approximately 7% of ischemic strokes. The incidence of other etiologies is not well documented and is primarily known from case reports [15].

A thorough history, adapted to the patient’s age, is essential to guide the diagnostic approach by focusing on cardiovascular risk factors, immunodeficiency, and associated symptoms. Our patient indeed presented multiple cardiovascular risk factors, including tobacco use, obesity, and dyslipidemia. Neuroimaging remains pivotal in identifying the underlying etiology.

In the case of ischemic causes, even a delay of a few hours in diagnosis and treatment can have serious consequences for the patient's functional prognosis. To exclude or promptly treat a cerebrovascular event, any presentation suggestive of MGS warrants urgent brain imaging. In our case, while brain CT was the most accessible initial modality, it was non-diagnostic. When a brain MRI is not immediately available, a brain CT remains the first-line examination to exclude intracranial hemorrhage (a formal contraindication to thrombolysis). Brain MRI is the imaging modality of choice for diagnosis and characterization of pontine lesions [16].

In our patient’s case, an NIHSS score < 4 without a significant neurological deficit did not meet the criteria for thrombolysis. Nevertheless, she received standard treatment for minor ischemic strokes not treated with thrombolysis, namely dual antiplatelet therapy [17].

## Conclusions

This case underscores the importance of performing a thorough neurological and clinical examination in patients presenting with seemingly benign peripheral facial paralysis. When associated with contralateral motor deficits, the condition is consistent with MGS, typically caused by a pontine lesion, most frequently ischemic in older individuals. It mandates urgent medical evaluation and management.
